# Catalytic hydrolysis of agar using magnetic nanoparticles: optimization and characterization

**DOI:** 10.1186/s13068-023-02441-w

**Published:** 2023-12-13

**Authors:** Anoth Maharjan, Wonho Choi, Hee Taek Kim, Jung-Ho Park

**Affiliations:** 1 Bio-Evaluation Center, Korea Research Institute of Bioscience and Biotechnology, Cheongju, 28116 Republic of Korea; 2 4D Convergence Technology Institute (National Key Technology Institute in University), Korea National University of Transportation, Jungpyeong, 27909 Republic of Korea; 3 Department of Food Science and Technology, Chungnam National University, Daejeon, 34134 Republic of Korea; 4 Department of Biosystems and Bioengineering, KRIBB School of Biotechnology, Korea University of Science and Technology (UST), 217 Gajeong-Ro, Yuseong-Gu, Daejeon, Korea

**Keywords:** Magnetic nanoparticles, Optimization, Hydrolysis, Agar, Bioethanol

## Abstract

**Background:**

Agar is used as a gelling agent that possesses a variety of biological properties; it consists of the polysaccharides agarose and porphyrin. In addition, the monomeric sugars generated after agar hydrolysis can be functionalized for use in biorefineries and biofuel production. The main objective of this study was to develop a sustainable agar hydrolysis process for bioethanol production using nanotechnology. Peroxidase-mimicking Fe_3_O_4_-MNPs were applied for agar degradation to generate agar hydrolysate-soluble fractions amenable to *Saccharomyces cerevisiae* and *Escherichia coli* during fermentation.

**Results:**

Fe_3_O_4_-MNP-treated (Fe_3_O_4_-MNPs, 1 g/L) agar exhibited 0.903 g/L of reducing sugar, which was 21-fold higher than that of the control (without Fe_3_O_4_-MNP-treated). Approximately 0.0181% and 0.0042% of ethanol from 1% of agar was achieved using *Saccharomyces cerevisiae* and *Escherichia coli*, respectively, after process optimization. Furthermore, different analytical techniques (FTIR, SEM, TEM, EDS, XRD, and TGA) were applied to validate the efficiency of Fe_3_O_4_-MNPs in agar degradation.

**Conclusions:**

To the best of our knowledge, Fe_3_O_4_-MNP-treated agar degradation for bioethanol production through process optimization is a simpler, easier, and novel method for commercialization.

## Introduction

Agar is the key component of the cell walls of certain red algae, such as *Gelidium* and *Garcilaria* [1, 2], which is composed of the polysaccharides agarose and porphyrin [3]. Porphyran consists of a porphyrobiose repeating unit (G-L6S) of -l-galactose-6-sulfate, whereas agarose is a linear polysaccharide composed of an agarobiose repeating unit (G-AHG) of -d-galactose and -l-galactose-3,6-anhydro [4, 5]. Agar is subsequently used as a gelling agent [6] that possesses a variety of biological properties, such as in microbial cultivation and vegetable tissue culture, and may be employed in a wide range of commercial applications, including the food, cosmetics, and pharmaceutical sectors [7]. In addition, agar monomeric sugars, such as d-galactose, 3,6-anhydro-l-galactose, and l-galactose-6-sulfate, can be functionalized for a variety of biorefineries or biofuel generation [8].

To address a variety of challenges, including the depletion of fossil fuel reserves and greenhouse gas (GHG) emissions, biofuels, such as bioethanol obtained from the synthesis of algal biomass, may be considered a practical substitute for fossil fuels [9–11]. Compared with lignocellulosic biomass, algal biomass (agar) comprises a high concentration of polysaccharides and lipids free of lignin, easing hydrolysis [12, 13]. Although biofuel production using the agar substrate is the most eco-friendly process, the difficulties associated with it must be avoided for industrial use. The hysteresis properties of agar complicate its use, which has been addressed using different approaches, including physiochemical pretreatment processes, such as microwave-assisted hydrothermal technology [14], deep eutectic solvent (DES) [15], alkaline pretreatment [16], acid catalyst pretreatment [17], synthetic biology [18], and genetic engineering [5]. Because it solidifies with adding water, chemical liquefication and enzymatic saccharification can be alternatives for generating fermentable monosugars. Different physiochemical techniques have been employed to degrade the algal polymer structure to liberate the fermentable monosugars for subsequent enzymatic hydrolysis [19–22] by releasing inhibitory compounds, such as hydroxymethyl furfural (HMF), a phenolic molecule that can prevent microbial fermentation [23, 24]. Meanwhile, the use of biological pretreatments, such as microbial strains and enzymes for agar degradation, is preferable, because it requires less energy, produces no inhibitors, and requires ambient working conditions [25]. However, biological approaches have several limitations, such as a high cost, limited necessity for biocatalysts, and stability. These difficulties with the physicochemical and biological agar degradation methods limit their use in the development of eco-friendly and successful pretreatment methods for agar feedstocks [26].

Owing to their distinctive characteristics, such as large surface area, high surface to volume ratio, ease of isolation, and electro conductivity, nanoparticles (NPs) have recently been used as substitutes for enzyme-mimicking behavior (also known as nanozymes) in a variety of fields, including agriculture, biofuels, and biomedicine [27]. It is possible to alter the size, shape, and doping of enzyme-mimicking NPs to modify their properties. Several nanomaterials (Co_3_O_4_, Fe_3_O_4_, Pt, Ag, Au, Zn, CeO_2_) have been identified for enzyme-mimicking activities, including those of peroxidase, oxidase, and catalase [28]. Fe-based NPs have demonstrated distinctive enzyme-mimicking properties and magnetic behaviors [29]. Pena et al*.* executed two separate magnetic nanoparticles (MNPs) incorporating different acid functions to demonstrate the considerable catalytic hydrolysis of wheat straw [30].

Considering the aforementioned, the synthesized Fe_3_O_4_-MNPs were employed for the pretreatment of agar substrates in the presence of hydrogen peroxidase (H_2_O_2_) and bioethanol fermentation in this study. *Escherichia coli* and *Saccharomyces cerevisiae* strains were used for bioethanol production through the consolidated bioprocessing of pretreated agar, which significantly reduced the overall cost of the agar substrate for bioethanol production. Many studies related to bioethanol production have been conducted using carbohydrate sources present in algal biomass [31]. Various structural characterization methods, such as Fourier transform infrared (FTIR), field emission scanning electron microscopy (FESEM), transmission electron microscopy (TEM), energy dispersive spectroscopy (EDS), X-ray power diffraction (XRD), and thermogravimetric analysis (TGA) of the untreated and Fe_3_O_4_-MNP-treated agar were utilized to validate the efficiency of the Fe_3_O_4_-MNPs-mediated degradation of agar in a sustainable manner. Furthermore, a central composite design (CCD) for the optimization process was applied to determine various operating parameters. An analysis of variance was used to determine the individual and interaction effects of these parameters. Thus far, several studies have been reported regarding bioethanol fermentation using nanomaterials in several steps. However, in this study, Fe_3_O_4_-MNPs were used for pretreatment, followed by fermentation without enzymatic saccharification, thereby reducing the use of enzymes. To the best of our knowledge, the application of agar pretreatment using Fe_3_O_4_-MNPs without enzymes through process optimization (CCD) for bioethanol production has not been reported relative to the current development of biorefineries. The use of nanoparticles for the hydrolysis of agar for bioethanol production is considerably feasible and can decrease the environmental impact, as NPs can be extracted from marine sources, such as seaweed.

## Results and discussion

### Estimation of the nanoparticle concentration, hydrogen peroxide concentration, and effect of temperature on reducing the sugar production

**Fig. 1:**
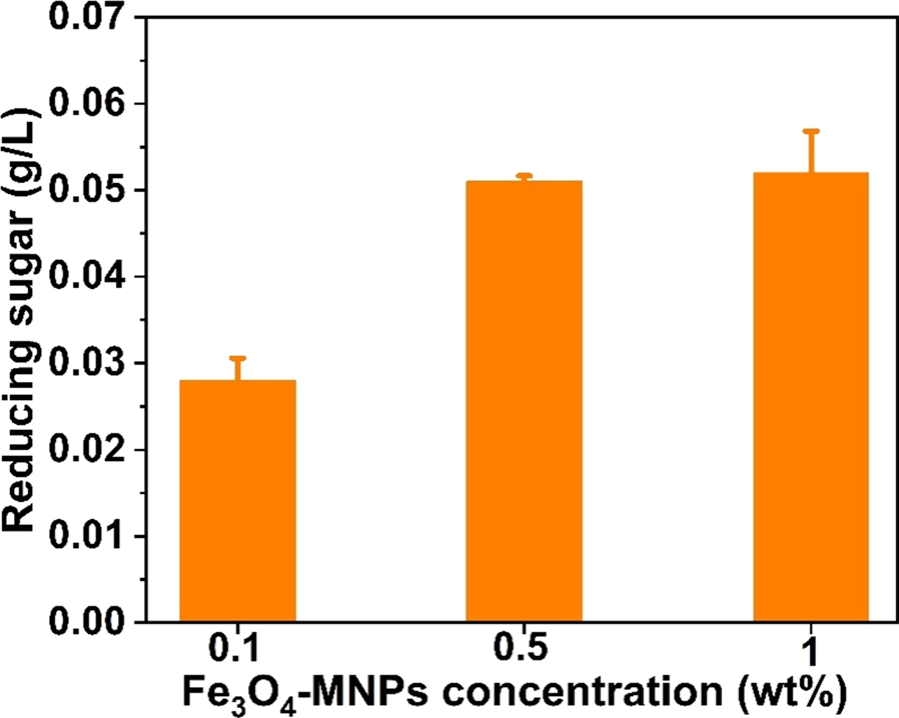
Comparison on the reducing sugar production when different concentration (0.1%, 0.5%, and 1%) of Fe_3_O_4_-MNPs was used in presence of distilled water (DW) as catalytic solvent

**Fig. 2:**
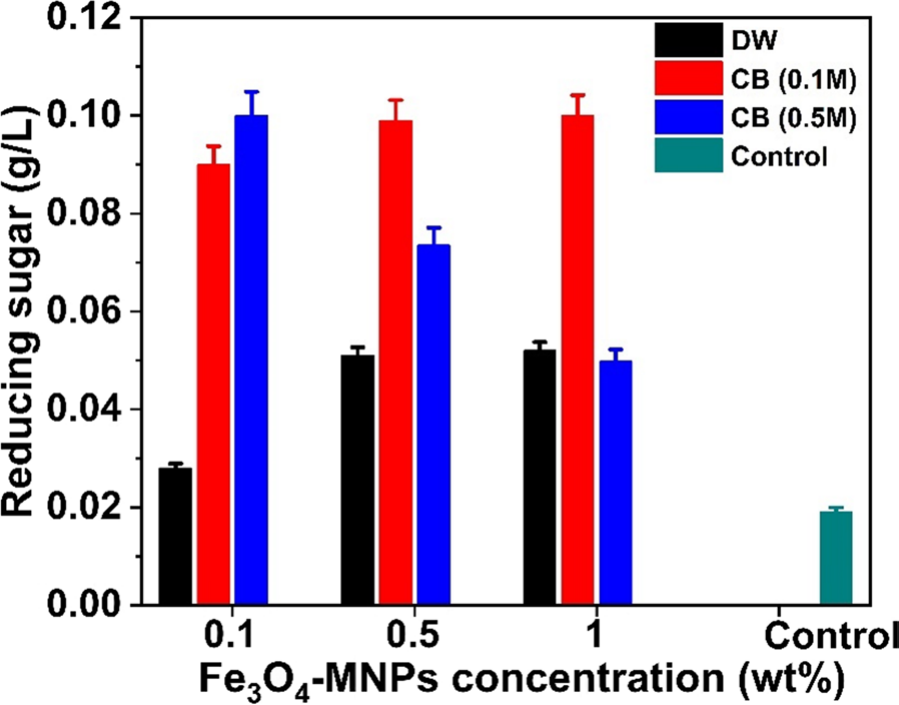
Comparison on the reducing sugar production with (0.1%, 0.5%, and 1%) Fe_3_O_4_-MNPs and without (Control) Fe_3_O_4_-MNPs in presence of distilled water (DW) and citrate buffer (CB, pH6.2), respectively

**Fig. 3:**
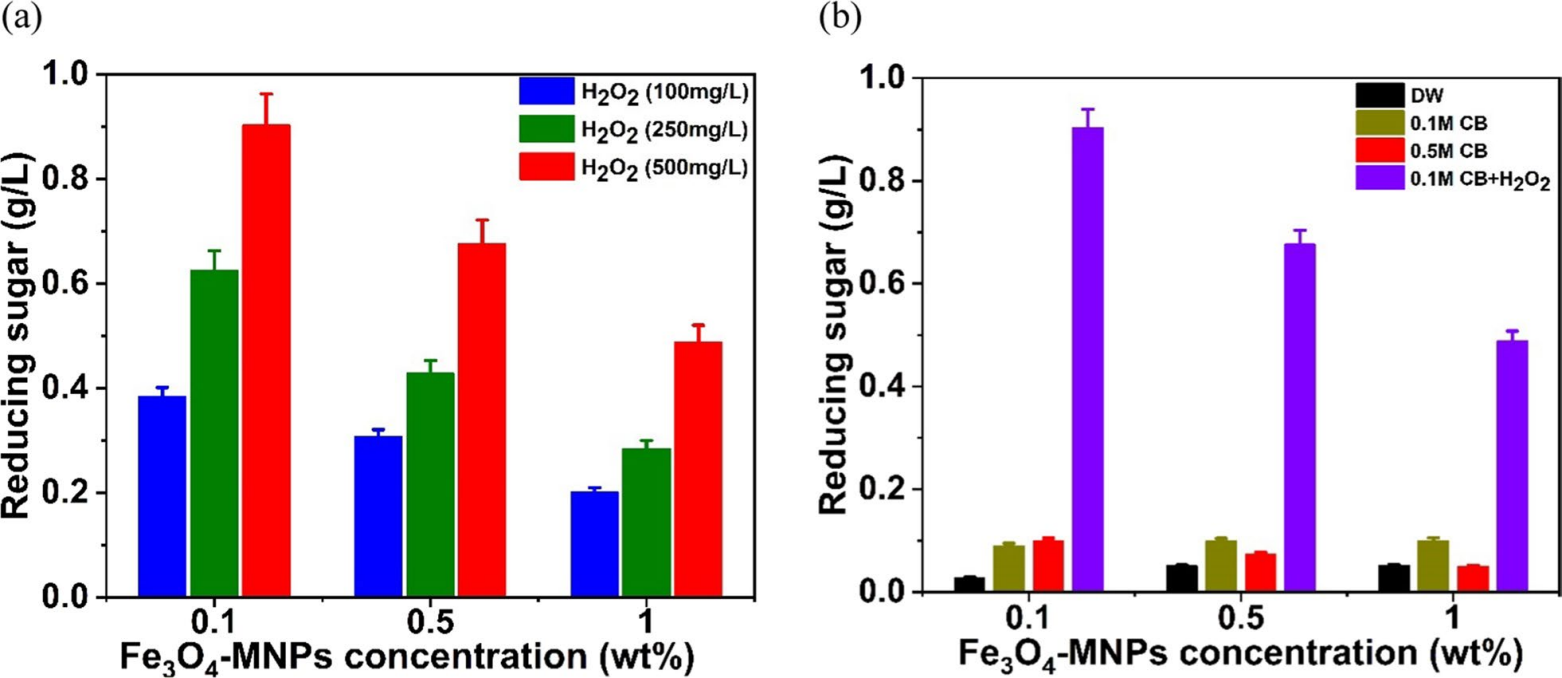
Estimation of reducing sugar production when different concentration (100, 250, and 500 mg/L) of hydrogen peroxide was used (**a**) and with the combination of citrate buffer (CB) and 500 mg/L hydrogen peroxide (**b**)

**Fig. 4:**
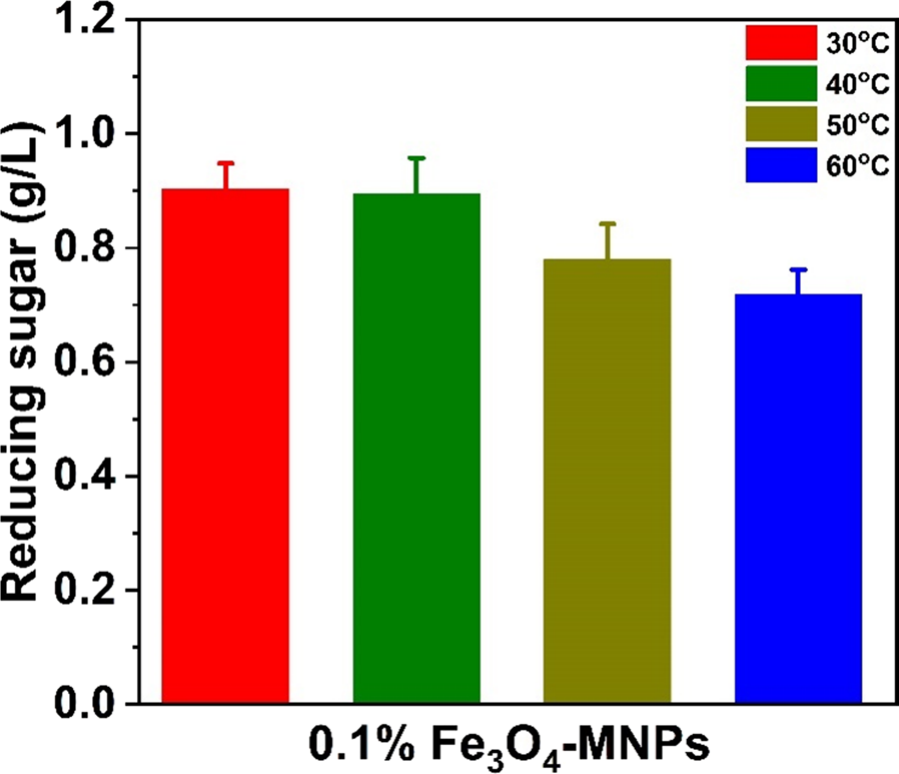
Illustration on reducing sugar production at different (30, 40, 50, and 60 °C) temperatures

First, the concentration of nanoparticles in high-reducing sugar products was evaluated using various MNP concentrations (0.1%, 0.5%, and 1%). At each of these three concentrations, 1% of MNPs produced 0.052 g/L of reducing sugar from 10 g/L (1%) agar when only dH_2_O was used (Fig. 1). In addition, citrate buffer (CB pH 6.2) at two different concentrations (0.1 and 0.5 M) was employed to improve the synthesis of reducing sugars (Fig. 2). Notably, approximately 0.1 g/L (1.9-fold) of reducing sugar was achieved when 0.1 M of citrate buffer (pH 6.2) along with 0.1% of MNPs were used, whereas it decreased when 0.5% and 1% of MNPs were used, as illustrated in Fig. 2. Nanoparticles at higher concentrations are difficult to distribute in water owing to their ferromagnetic characteristics (Fe_3_O_4_-MNPs) and require many washing processes for further saccharification of the pretreated biomass [32]. Therefore, a higher concentration of nanoparticles (Fe_3_O_4_-MNPs) for pretreatment is not feasible on an industrial scale. Considering different concentrations of hydrogen peroxide (100, 250, and 500 mg/L) along with 0.1 M CB (pH6.2) (Fig. 3a), nearly 0.903 g/L (21-fold) of reducing sugar was obtained when 500 mg/L hydrogen peroxide was used (Fig. 3b). Hydrogen peroxidase can generate free radicals in the presence of hydrogen peroxidase, which help in the degradation of agar to release fermentable sugar. Finally, the effects of temperature (30, 40, 50, and 60ºC) on reducing the sugar production were examined using the aforementioned combinations. Among these, a high-reducing sugar was produced at 30 °C, as shown in Fig. 4. Based on these results, we designed the experiments using the response surface methodology (RSM) for bioethanol production.

### Optimization of bioethanol production and its interaction using RSM

Bioethanol production was optimized using RSM, where a central composite design (CCD) full factorial design matrix, each with 20 individual runs for *Escherichia coli* K12 and *Saccharomyces cerevisiae* S288c ATCC 7754, is provided in Table 1a, b, respectively. Based on the response values, the bioethanol production ranged from 0.0018% (run no. 4) to 0.0042% (run no. 3) for *Escherichia coli* K12, and 0.002% (run no. 17) to 0.181% (run no. 1) for *Saccharomyces cerevisiae* S288c ATCC 7754. The response variables that corresponded to the coded values of the variables were adjusted to the quadratic model, as provided in Eq. (4). The linear, square, and interaction coefficients with coded *p* and *t* values are shown in Tables 2a for *Escherichia coli* K12 and (b) for *Saccharomyces cerevisiae* S288c ATCC 7754. The derived quadratic polynomial equation in terms of the coded variables, which characterizes the interaction between the independent variables and the response factor, is as follows:

1 $$ {\text{For}}\;Escherichia \, coli{:}\;Y_{bioethanol\;production\;(\% )} \; = \;{0}{\text{.0263}}\;{ + }\;{0}{\text{.0000015}}X_{1}^{2} \; - \;{0}{\text{.000019}}X_{2}^{2} { + 0}{\text{.000012}}X_{3}^{2} $$

2 $$ \begin{aligned} {\text{For}}\;Saccharomyces \, cerevisiae{:}\;Y_{bioethanol\;production\;(\% )} \; = \; & 0.0{59}{-}0.00{112}X_{1} - \, 0.00{23}X_{2} \\ + & \;0.00{34}X_{3} + \, 0.0000{18}X_{1} X_{2} \\ \end{aligned} $$

where *Y* denotes the bioethanol production (%), and *X1*, *X2*, and *X3* are the coded values of the independent variables, as shown in Table 6.

**Table 1:** Central composite design matrix of optimization variables with actual values and response variable for ethanol production

Run	Hydrolysate concentration (%)	Temperature ºC	Time H	Ethanol concentration (%)	
				Actual value	Predicted value
(a)* Escherichia coli* K12					
1	1.47759	33.5	5.5	0.0023	0.0026
2	22.5	33.5	5.5	0.0020	0.0020
3	35	30	8	0.0042	0.0040
4	10	37	3	0.0018	0.0016
5	22.5	33.5	5.5	0.0020	0.0020
6	22.5	33.5	5.5	0.0020	0.0020
7	22.5	33.5	1.29552	0.0020	0.0023
8	22.5	33.5	9.70448	0.0019	0.0022
9	10	37	8	0.0021	0.0019
10	22.5	27.6137	5.5	0.0023	0.0025
11	43.5224	33.5	5.5	0.0025	0.0028
12	10	30	3	0.0035	0.0033
13	35	37	8	0.0023	0.0021
14	35	30	3	0.0024	0.0022
15	22.5	39.3863	5.5	0.0025	0.0028
16	10	30	8	0.0027	0.0025
17	22.5	33.5	5.5	0.0020	0.0020
18	22.5	33.5	5.5	0.0020	0.0020
19	22.5	33.5	5.5	0.0020	0.0020
20	35	37	3	0.0027	0.0025
(b) *Saccharomyces cerevisiae* S288c (ATCC 7754)					
1	10	30	8	0.0181	0.0163
2	22.5	35	5.5	0.0056	0.0056
3	35	40	8	0.0022	0.0027
4	22.5	35	9.7	0.0071	0.0071
5	22.5	35	1.29	0.0046	0.0043
6	22.5	35	5.5	0.0056	0.0056
7	22.5	43.409	5.5	0.0054	0.0033
8	10	40	3	0.0064	0.0065
9	35	30	8	0.0072	0.0073
10	43.5224	35	5.5	0.0051	0.0043
11	10	30	3	0.0119	0.0116
12	22.5	35	5.5	0.0056	0.0056
13	22.5	26.591	5.5	0.0097	0.0115
14	22.5	35	5.5	0.0056	0.0056
15	22.5	35	5.5	0.0056	0.0056
16	1.47759	35	5.5	0.0135	0.014
17	35	40	3	0.002	0.004
18	10	40	8	0.0059	0.0072
19	22.5	35	5.5	0.0056	0.0056
20	35	30	3	0.0058	0.0047

**Table 2:** Results of central composite design for ethanol concentration

Model term	Coefficient estimate	Computed *t *value	*p* Value
(a) Model coefficients, *t* and *p* value for second-order regression model for *Escherichia coli* K12			
Intercept (*β*_*0*_)	0.001986	8.73	0.000*
* Linear coefficients*			
*X*_1_ (Hydrolysate concentration %)	0.000226	0.89	0.394
*X*_2_ (Temperature ºC)	− 0.000439	*− *1.73	0.115
*X*_3_ (Time H)	0.000090	0.35	0.730
* Square coefficients*			
*X*_1_^2^ (Hydrolysate concentration % x Hydrolysate concentration %)	0.000667	1.60	0.140
*X*_2_^2^ (Temperature ºC x Temperature ºC)	0.000667	1.60	0.140
*X*_3_^2^ (Time H x Time H)	0.000217	0.52	0.613
*Interaction coefficients*			
*X*_1_*X*_2_ (Hydrolysate concentration % x Temperature ºC)	0.000247	0.44	0.667
*X*_1_*X*_3_ (Hydrolysate concentration % x Time H)	0.000672	1.20	0.256
*X*_2_*X*_3_ (Temperature ºC x Time H)	*− *0.000389	*− *0.70	0.502
(b) Model coefficients, *t* and *p* value for second-order regression model for *Saccharomyces cerevisiae* S288c (ATCC 7754)			
Intercept (*β*_*0*_)	0.005606	9.93	0.000 *
* Linear coefficients*			
*X*_1_ (Hydrolysate concentration %)	*− *0.004829	*− *7.67	0.000 *
*X*_2_ (Temperature ºC)	*− *0.004151	*− *6.59	0.000 *
*X*_3_ (Time H)	0.001417	2.25	0.048 *
* Square coefficients*			
*X*_1_^2^ (Hydrolysate concentration % x Hydrolysate concentration %)	0.00355	3.45	0.006 *
*X*_2_^2^ (Temperature ºC x Temperature ºC)	0.00180	1.75	0.111
*X*_3_^2^ (Time H x Time H)	0.00010	0.10	0.922
* Interaction coefficients*			
*X*_1_*X*_2_ (Hydrolysate concentration % x Temperature ºC)	0.00315	2.27	0.046 *
*X*_1_*X*_3_ (Hydrolysate concentration % x Time H)	*− *0.00145	*− *1.05	0.319
*X*_2_*X*_3_ (Temperature ºC x Time H)	*− *0.00279	*− *2.02	0.071

### Analysis of variance (ANOVA)

Model significance in quadratic terms was estimated using the *t* test, *F* value, and *p* value. A higher *t* test value indicates a greater significant coefficient of the model, whereas a *p *value < *0.05* represents the excellent significance of the response variables. Table 3 presents the ANOVA results of the quadratic models. In addition, the *F* values (4.71 for *Escherichia coli* K12 and 14.64 for *Saccharomyces cerevisiae* S288c ATCC 7754) and *p* values (0.00001) indicated that the model was highly significant. The correlation coefficient (*R*^*2*^) value of 0.90 for *Escherichia coli* K12 and 0.92 for *Saccharomyces cerevisiae* S288c ATCC 7754 indicated a similarity between the experimental and predicted values of the response; greater *R*^*2*^ values affirm the model.

**Table 3:** Estimation of analysis of variance for regression expression

Source	DF	SS	MS	*f* value	*p* value
(a) ANOVA for quadratic model *Escherichia coli* K12					
Regression	13	0.000003	0.000000	4.17	0.0447*
*X*_1_ (Hydrolysate concentration %)	1	0.000000	0.000000	0.1863	0.6811
*X*_2_ (Temperature ºC)	1	0.000001	0.000001	0.2054	0.6663
*X*_3_ (Time H)	1	0.000000	0.000000	0.0466	0.8363
*X*_1_^2^	1	0.000001	0.000001	2.57	0.0339*
*X*_2_^2^	1	0.000001	0.000001	2.57	0.0348*
*X*_3_^2^	1	0.000000	0.000000	0.27	0.04061*
*X*_1_*X*_2_	1	0.000000	0.000000	0.20	0.667
*X*_1_*X*_3_	1	0.000000	0.000000	1.45	0.256
*X*_2_*X*_3_	1	0.000000	0.000000	0.49	0.502
Residual** (**Error)	10	0.000003	0.000000		
Lack-of-Fit	1	0.000003	0.000001	*	*
Pure Error	5	0.000000	0.000000		
Total	19	0.000006			
(b) ANOVA for quadratic model *Saccharomyces cerevisiae* S288c (ATCC 7754)					
Regression	9	0.00003	0.000000	14.64	0.0001*
*X*_1_ (Hydrolysate concentration %)	1	0.00001	0.00001	58.82	< 0.0001*
*X*_2_ (Temperature ºC)	1	0.00001	0.00001	43.45	< 0.0001*
*X*_3_ (Time H)	1	0.00001	0.00001	5.06	0.048*
*X*_1_^2^	1	0.000000	0.000000	11.87	0.006*
*X*_2_^2^	1	0.000006	0.000006	3.05	0.111
*X*_3_^2^	1	0.000000	0.000000	0.01	0.8854
*X*_1_*X*_2_	1	0.00001	0.00001	5.17	0.046*
*X*_1_*X*_3_	1	0.000002	0.000002	1.10	0.319
*X*_2_*X*_3_	1	0.000008	0.000008	4.08	0.071
Residual (Error)	10	0.000019	0.000002		
Lack-of-Fit	5	0.000019	0.000004	*	*
Pure Error	5	0.000000	0.000000		
Total	19	0.0002	0.000000	14.40	0.0001*

DF: degree of freedom, SS: sum of square, and MS: mean square

*Significant *p* ≤ 0.05

### Process variables through interaction effects

**Fig. 5.**
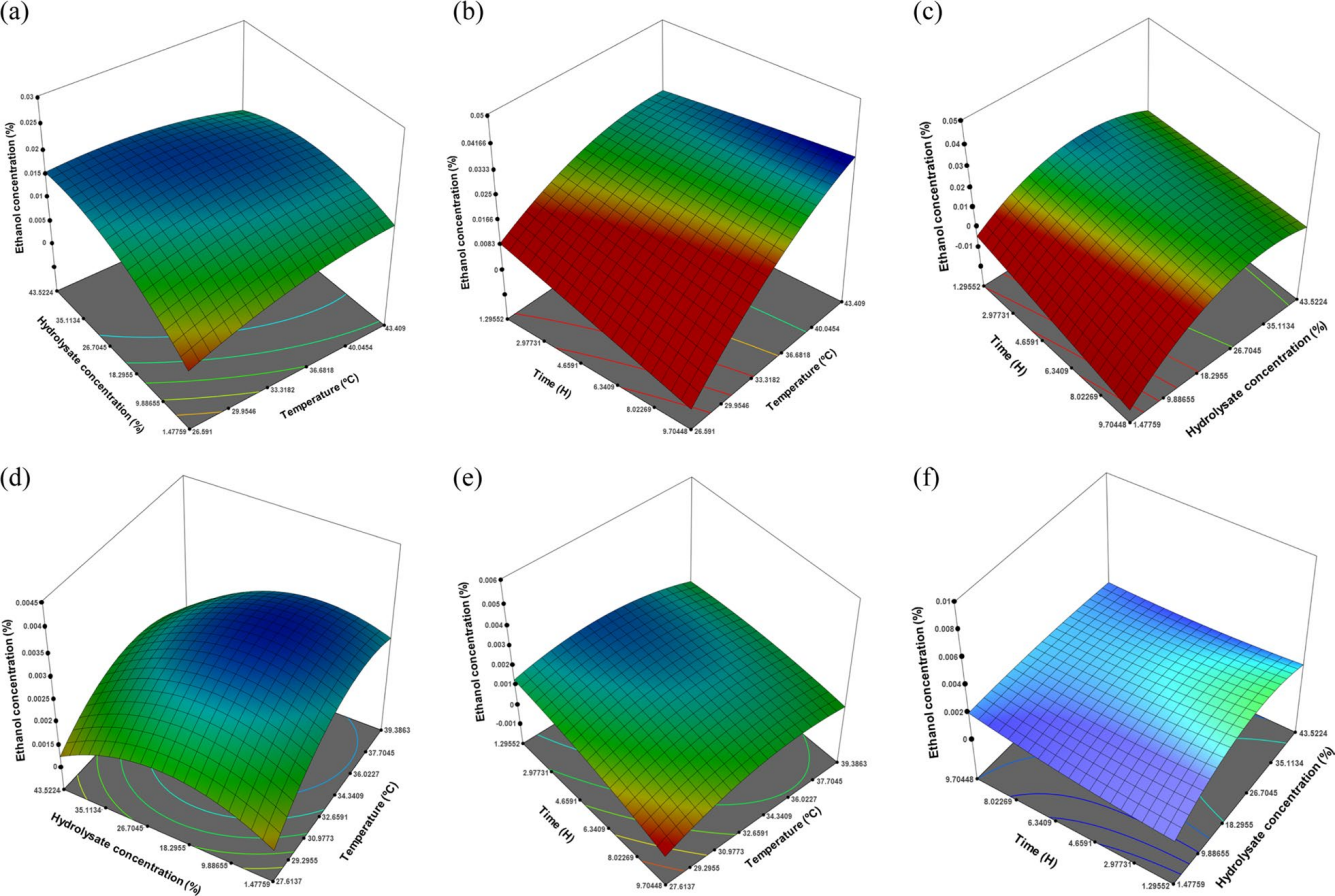
3D plots depicting interactions among different process variables; (**a**, **d**) hydrolysate concentration (%), temperature (^o^C); (**b**, **e**) time (H), temperature (^o^C) and (**c**, **f**) time (H), hydrolysate concentration (%) for both *Saccharomyces cerevisiae* and *Escherichia coli*

A 3D surface plot illustrates the synergistic effect of the two process variables on the efficiency of bioethanol production (Fig. 5). An increase in the hydrolysate concentration and temperature resulted in an increase in the bioethanol production efficiency for *Saccharomyces cerevisiae* S288c ATCC 7754 (Fig. 5a), whereas considering time and temperature (Fig. 5b), increases in both response variables exhibited a positive influence on the bioethanol production efficiency. Similarly, an increase in the reaction time and hydrolysate concentration also elucidated an increase in the bioethanol production efficiency, as shown in Fig. 5c. Overall, an increase in the process variables positively influenced bioethanol production.

For *Escherichia coli* K12, an increase in the hydrolysate concentration and temperature resulted in an increase in the bioethanol production efficiency up to a certain point, followed by a decrease in the bioethanol production with a further increase in the hydrolysate concentration (Fig. 5d). Similarly, the interaction between the time and temperature is illustrated in Fig. 5e. An increase in the reaction time resulted in a decrease in the bioethanol concentration, whereas an increase in temperature resulted in an increase in the bioethanol concentration. Furthermore, the effects of the reaction time and hydrolysate concentration are illustrated in Fig. 5f. The bioethanol production efficiency increased as the hydrolysate concentration and reaction time increased. This indicates that an increase in temperature enhanced the overall bioethanol concentration. In addition, a higher hydrolysate concentration may lead to exhaustion of the carbon source, leading to continuous bacterial growth.

Optimization of the process variables for bioethanol production using *Saccharomyces cerevisiae* S288c (ATCC 7754) and *Escherichia coli* K12 was conducted (as depicted in Table 1a, b), which demonstrated that when *Saccharomyces cerevisiae* S288c (ATCC 7754) was used, bioethanol production was comparatively higher than *Escherichia coli K12*. This is because *Saccharomyces cerevisiae* S288c (ATCC 7754) is known to utilize galactose and glucose, which is formed sequentially from agar hydrolysis, and can also tolerate a wide range of pH values [33, 34], whereas *Escherichia coli* K12 can only utilize glucose. Ethanol production using the synthesized Fe_3_O_4_-MNPs was comparatively lower (Table 4) than the simultaneous enzymatic hydrolysis. Not using bacterial enzymes for the hydrolysis of agar results in a low ethanol yield, despite the simultaneous saccharification of biomass with enzymes enhancing the reducing sugar production, as reported in other studies [35–37]. However, considering the cost limitations, which are assumed to account for approximately 20% of the ethanol production costs [36], slow reaction rate resulting in a time-consuming hydrolysis, and difficulty in enzyme recovery, the enzymatic hydrolysis process remains under consideration [38, 39]. Byproducts formed during hydrolysis, such as furfural, HMF, and formic acid, inhibit enzymatic activity [40, 41]. In addition, enzymatic degradation may occur after thermal hydrolysis, leading to researchers focusing on developing thermostable enzymes [42]. Therefore, the use of the synthesized Fe_3_O_4_-MNPs for bioethanol production can be a new and feasible method. Using synthesized Fe_3_O_4_-MNPs in the absence of bacterial cells has a limited hydrolysis potential for agar, which ultimately reduces the release of monosugars. However, considering certain other prospective studies, using only Fe_3_O_4_-MNPs can be a new strategy to overcome the drawbacks possessed by other (chemical, hydrothermal, and enzymatic) hydrolysis techniques.

**Table 4:** Comparison of ethanol yield using different hydrolysis technique in presence of different substrates

Feedstock	Config	Microorganisms	EtOH	References
*U. fasciata* (green)	EH, SHF	*Saccharomyces cerevisiae* MTCC 180	47 (g/100 g)	[68]
*U. fasciata* (green)	EH, SHF	*Saccharomyces cerevisiae*	47 (g/100 g)	[69]
*Gracilaria* sp. (red)	AH & EH, SHF	*Saccharomyces cerevisiae* Wu	47 (g/100 g)	[70]
*G. amansii* (red)	AH, SHF	*Brettanomyces custersii*	38 (g/100 g)	[71]
*L. japonica* (brown)	AH & EH, SHF	*Escherichia coli* KO11	41 (g/100 g)	[72]
*K. alvarezii* (red)	AH, SSF	Brewer’s yeast	21 (g/100 g)	[73]
*Sargassum sagamianum* (brown)	TH & EH, SHF	*P. stipites* CBS7126	44 (g/100 g)	[74]
*E. globulus*	SSF	*Saccharomyces cerevisiae* IR2T9	30–38 (g/L)	[75]
NS	CBP	*Saccharomyces cerevisiae* MT8-1	0.71 (g/L)	[76]
Raw CC	SPS using Ce–Fe_3_O_4_, EH	*Saccharomyces cerevisiae*	21.7 g/L	[77]
Recycled paper sludge	Batch/SHF	*Saccharomyces cerevisiae* PE-2	5.6–6.3 g/L	[78]
*E. globulus*	SSF	*Saccharomyces cerevisiae* D5A	5.67 g/L	[79]
Sugarcane bagasse	Phosphoric acid pretreatment	*Escherichia coli* MM170	0.25–0.27 (g/g raw biomass)	[80]
Lodgepole pine	SPORL pretreatment	NS	0.22 (g/g raw biomass)	[81]
Birch	Alkaline	NS	0.11 (g/g raw biomass)	[82]
*Miscanthus*	LHW	NS	0.15 (g/g raw biomass)	[83]
Agar	SPS using Fe_3_O_4_-MNPs	*Saccharomyces cerevisiae* S288c (ATCC 7754)	0.0181%	This study
Agar	SPS using Fe_3_O_4_-MNPs	*Escherichia coli* K12	0.0042%	This study

EtOH: ethanol production, EH: enzymatic hydrolysis, AH: acid hydrolysis, TH: thermal hydrolysis, SHF: separate hydrolysis and fermentation, SSF: simultaneous saccharification and fermentation, CBP: consolidated bioprocessing, SPS: simultaneous pretreatment and saccharification, LHW: liquid hot water, SPORL: sulfite pretreatment to overcome recalcitrance of lignocellulose, CC: Corn cob, NS: not specified

### Experimental validation

The consistency of the model between the individual experiments and process optimization conditions were tested according to those predicted by the CCD analysis in response to the bioethanol concentration. Table 5 exhibits the optimum process conditions and bioethanol production, where the final bioethanol production obtained after the experiment run with the predicted variables was recorded as 0.003 for *Escherichia coli* K12 and 0.072 for *Saccharomyces cerevisiae* S288c ATCC 7754. The precise variable concentration for the bioethanol production efficiency was determined using process-variable optimization.

**Table 5:** Global optimum values of variables with predicted and actual response

Hydrolysate concentration (%)	Temperature (ºC)	Time (H)	Ethanol concentration (%)	
			Predicted	Actual
(a) *Escherichia coli* K12				
35	27.6	9.7	0.0225	0.003
(b) *Saccharomyces cerevisiae* S288c (ATCC 7754)				
1.47	26.6	9.7	0.225	0.072

### Characterization of Fe_3_O_4_-MNPs and agar

**Fig. 6:**
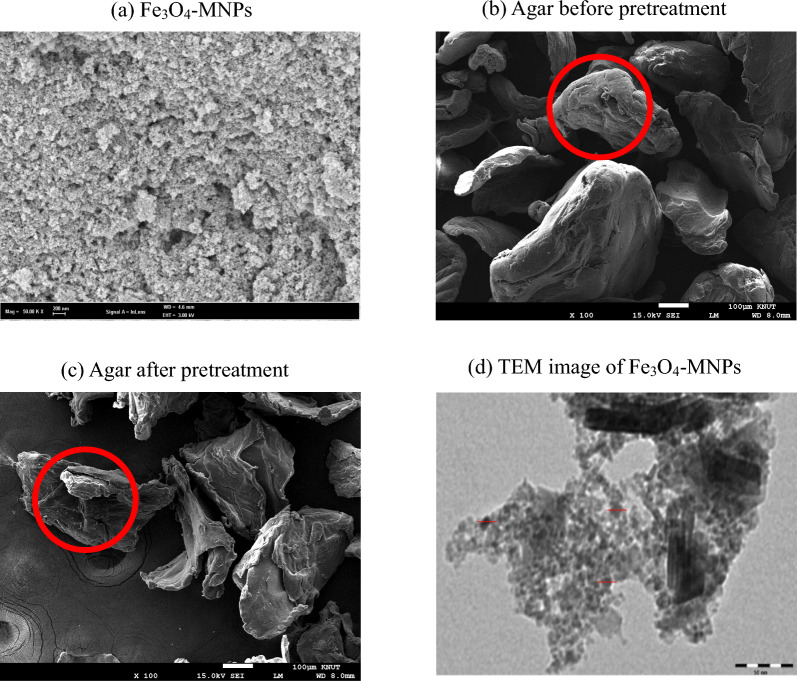
Morphology characterization of synthesized Fe3O4-MNPs. **a** SEM analysis of synthesized Fe_3_O_4_-MPNs, **b** SEM image of agar before pretreatment, **c** SEM image of agar after pretreatment with 0.1% Fe_3_O_4_-MNPs, 500 mg/L H_2_O_2_ and citrate buffer (CB) (pH6.2), and (**d**) TEM image of synthesized Fe_3_O_4_-MNPs

An SEM analysis was used to morphologically characterize Fe_3_O_4_-MNPs and agar before and after processing; the resulting images are displayed in Fig. 6a–c, respectively. As shown in Fig. 6a, the surface morphology of Fe_3_O_4_-MNPs demonstrates a spherical form [43]. Similarly, the agar surface morphology before pretreatment demonstrated a solid rigid structure (Fig. 6b); however, the surface morphology after pretreatment exhibited a disruption of the surface (Fig. 6c), because the magnetic iron nanoparticles (Fe_3_O_4_-MNPs) have the ability to produce reactive oxygen species (ROS) via the Fenton reaction from H_2_O_2_, causing oxidative stress and ultimately damaging the cell wall of the agar. In addition, TEM image of Fe_3_O_4_-MNPs exhibits a spherical shape with a uniform diameter in range of 8–10 nm (Fig. 6d) corelated with the reported one [44].

**Fig. 7:**
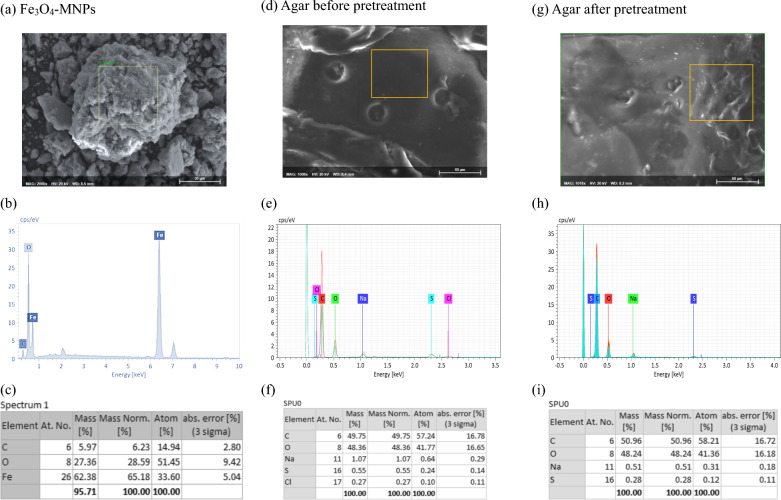
FESEM and EDS analysis of; (**a**–**c**) synthesized Fe_3_O_4_-MNPs, (**d**–**f**) agar before pretreatment, and (**g**–**i**) agar after pretreatment

Energy dispersive spectroscopy (EDS) and field-emission SEM were employed to determine the elemental composition of the synthesized Fe_3_O_4_-MNPs and agar before and after pretreatment (Fig. 7). Figure 7a–c presents the typical EDS spectra of the synthesized Fe_3_O_4_-MNPs with 62.38% Fe and 27.36% O, indicating that the primary components of the nanoparticles are iron and oxygen [32]. Similarly, Fig. 7d–f illustrates the EDS analysis of agar before pretreatment, demonstrating a maximum of 49.75% C and 48.36% O, and 50.96% C and 48.24% O. Likewise, Fig. 7g–i demonstrates the EDS analysis of agar after pretreatment, where the vanishing of Cl (Fig. 7i) indicates the C–O of carbohydrate in agar.

**Fig. 8:**
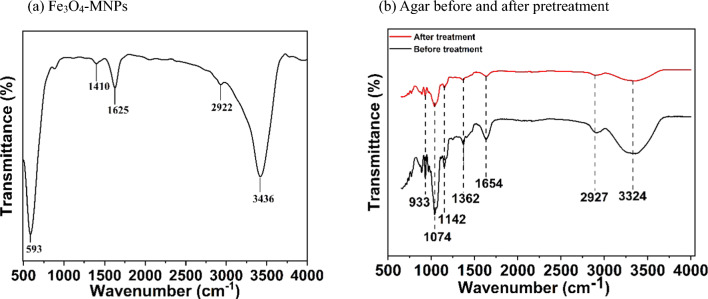
FTIR analysis of; (**a**) synthesized Fe_3_O_4_-MNPs, and (**b**) agar before and after pretreatment

The characteristic functional groups of the synthesized Fe_3_O_4_-MNPs and agar before and after pretreatment were determined by the FTIR analysis (Fig. 8a), which demonstrates a sharp peak at 593 cm^−1^ related to the stretching vibration of Fe–O from magnetite, which also denotes the purity of the nanoparticles [45]. Similarly, the broad peak at 3436 cm^−1^ corresponds to the –OH stretching vibration of the hydrogen bond of absorbed water on the surface of the nanoparticles [46]. The peak at 2922 cm^−1^ indicates the asymmetric stretching of C–H and the peak at 1625 cm^−1^ indicates the bending (*δ*) vibrations of the H─O─H groups [46, 47].

In Fig. 8b, the broad peak at 3324 cm^−1^ before and after pretreatment demonstrates the stretching vibration of –OH, indicating the presence of the hydroxyl group in agar [48], whereas after pretreatment, the peak shrinks, indicating the hydrolysis of agar. Furthermore, the peak at 2927 cm^−1^ before and after pretreatment corresponds to the C–H stretching vibrations of the –CH_3_ and –CH_2_ groups [49, 50]. The adsorption band at 1654 cm^−1^ attributed to the C = O and N–H stretching groups indicates the formation of conjugated peptides bonds [51]. In addition, the peak at 1362 cm^−1^ corresponds to the asymmetric bending stretching of –C–H to –CH_3_ [52], whereas the peaks at 1142 cm^−1^ and 1074 cm^−1^ correspond to the C–O–C stretching vibration of polysaccharides and the C–O stretching vibration of carbohydrates, respectively [53]. In addition, the peak at 933 cm^−1^ indicates the presence of a 3,6-anhydrous galactose bridge, confirming the agar composition [54, 55].

**Fig. 9:**
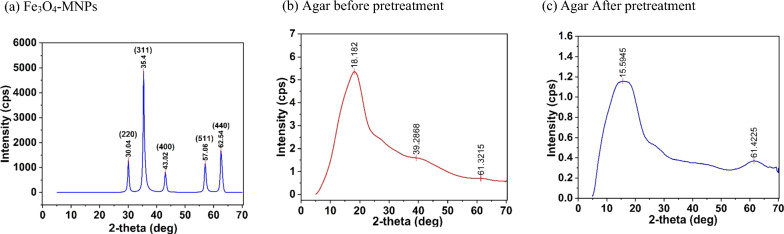
XRD characterization of; (**a**) synthesized Fe_3_O_4_-MNPs, (**b**) agar before pretreatment, and (**c**) agar after pretreatment

Figure 9 presents the XRD patterns of the synthesized Fe_3_O_4_-MNPs and agar before and after pretreatment. Regarding the diffraction planes of the Fe_3_O_4_-MNPs spinel structure, a succession of distinctive peaks was observed in the XRD pattern at 2θ of 30.04°, 35.4^o^, 43.02^o^, 57.06^o^, and 62.54^o^, corresponding to the diffraction planes of (220), (311), (400), (511), and (440), respectively, demonstrating the reflections of magnetite (Fig. 9a) and indicating the presence of the crystalline spinel-structured magnetite (Fe_3_O_4_-MNPs) phase of iron oxide, similar to that reported previously [56]. Similarly, the XRD pattern of agar before pretreatment indicating a peak at 2θ = 18.18^o^, 39.28^o^, and 61.32^o^ (Fig. 9b) defined the hydrated crystalline structure and accordingly, the presence of an amorphous structure [48]. In contrast, the broad peak shifted at 2θ = 15.59^o^ (Fig. 9c), indicating the increased release of the amorphous structures owing to the corrosion of the synthesized Fe_3_O_4_-MNPs, further promoting the release of reducing sugar.

**Fig. 10:**
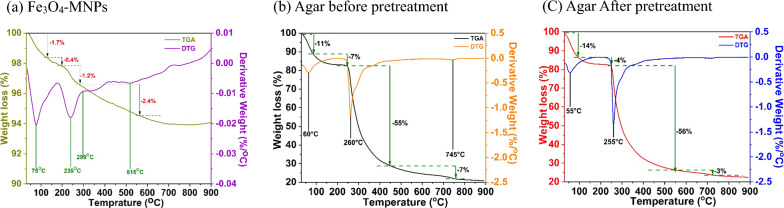
TGA and DTG characterization of (**a**) synthesized Fe_3_O_4_-MNPs, (**b**) agar before pretreatment, and (**c**) agar after pretreatment

A TGA analysis of the pyrolytic properties of the synthesized Fe_3_O_4_-MNPs and agar before and after pretreatment is shown in Fig. 10. The TGA curve of Fe_3_O_4_-MNPs shown in Fig. 10a demonstrates four phases of weight loss, where the first weight loss was observed between 30 and 130 °C with a mass loss of 1.7%. This was owing to the loss of the absorbed physical and chemical water from the surface of the NPs [57]. Furthermore, a second weight loss of 0.4% was observed between 130 and 200 °C, which corelated with the existence of certain combustible products in the sample. This loss of water from the sample was verified by the dips at 75 and 235 °C in the DTG curve. Similarly, a third weight loss of 1.2% was observed between 200 and 270 °C, resembling the complete rapid decomposition of the water residual as the first two steps. Finally, a 2.4% weight loss was observed at 270–560 °C, and as a dip at 515 °C in the DTG curve.

Regarding agar before the pretreatment, the first weight loss of 11% was observed between 30 and 100 °C, which was owing to the evaporation of free and bound water (Fig. 10b) [48]. Similarly, the weight loss of 7% in the second stage was observed owing to the remaining moisture in the sample. During the third stage, a maximum decomposition occurred with a weight loss of 55% between 270 and 450 °C. At this stage, higher molecular compounds such as carbohydrates, proteins, and lipids in the agar underwent cracking and depolymerization reactions owing to the continuous supply of heat [48]. This was verified by the dips at 260 and 745 °C in the DTG curve. Moreover, the third stage is considered an active pyrolytic zone, because it refers to the production of biofuels and is comparatively similar to that previously reported [58, 59].

Furthermore, Fig. 10c exhibits the pyrolytic properties of agar after pretreatment, where 14% of weight loss was observed between 30 and 100 °C followed by a 4% weight loss between 100 and 260 °C. Approximately 56% of weight loss was observed, which was slightly more than that before pretreatment (Fig. 10b) owing to the decomposition of higher compounds, such as polysaccharides, proteins, and lipids, due to the catalytic behavior of the synthesized Fe_3_O_4_-MNPs. At temperatures greater than 700 °C, no significant weight loss was observed, mostly owing to the presence of the inorganic content in the agar [60].

## Materials and methods

### Materials

Ferric chloride (FeCl_3_) and ferrous chloride (FeCl_2_) were purchased from Junsei Chemicals Co. Ltd., Japan. Hydrogen peroxide (H_2_O_2_, 34.5%) and the ammonia solution (20–35%) were purchased from Samchun Chemicals Co. Ltd., Korea. Sodium Citrate dihydrate (C_6_H_9_Na_3_O_9_. 2H_2_O) and Citric Acid (C_6_H_8_O_7_) (Sigma-Aldrich) were purchased, as well as Agar (Bacto™), LB broth, and the YM broth (Difco™).

### Synthesis of iron oxide MNPs (Fe_3_O_4_-MNPs)

**Fig. 11:**
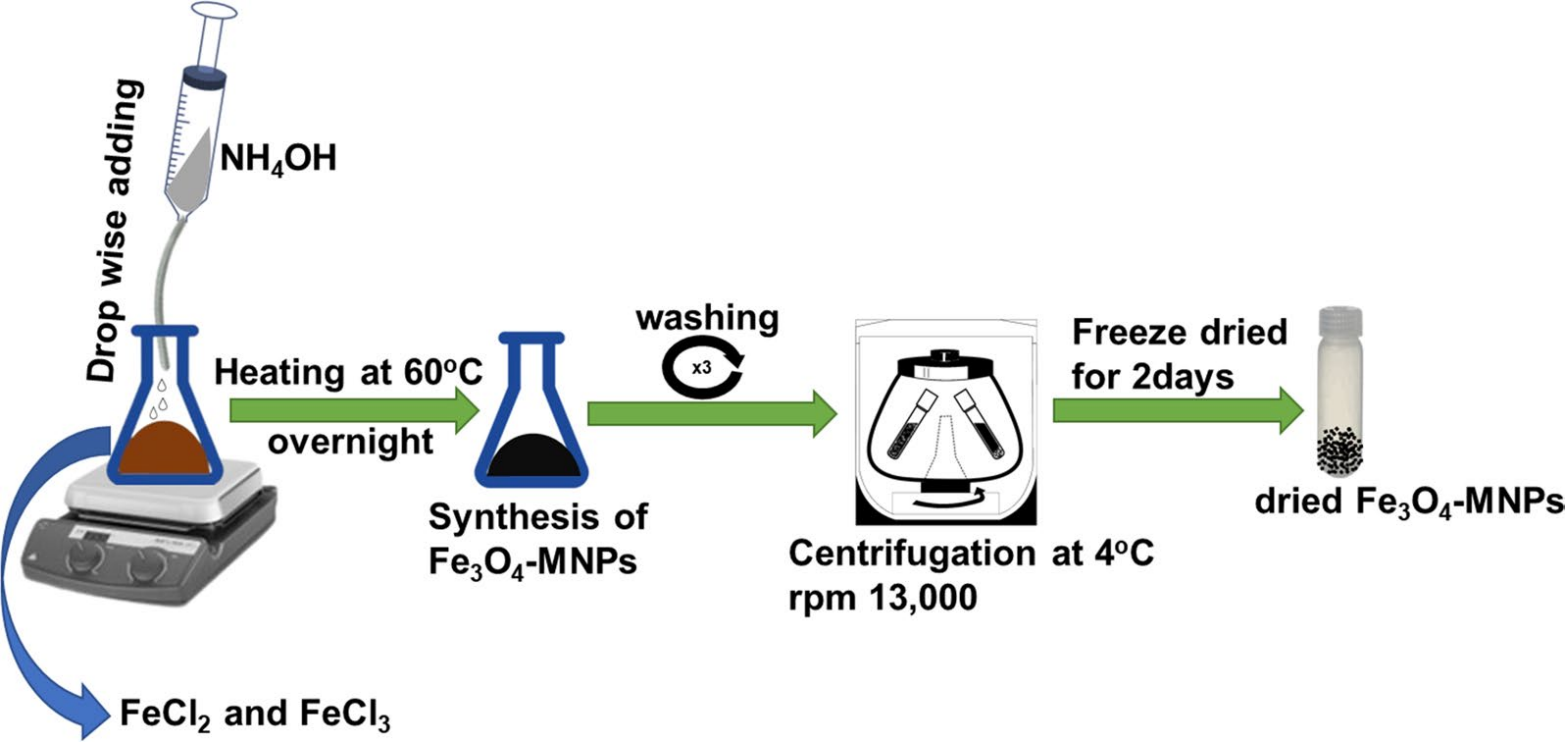
Schematic diagram of the chemically synthesis of magnetic iron nanoparticle (Fe_3_O_4_MNPs)

Fe_3_O_4_-MPNs were chemically synthesized, as described by Maharjan et al. [57], by combining 3 mol of ferric chloride (FeCl_3_) and 1 mol of ferrous chloride (FeCl_2_) in deionized water. Subsequently, an equal volume of ammonia solution was added dropwise and stirred at 60 ºC overnight (Fig. 11). The synthesized Fe_3_O_4_-MNPs were centrifuged at 15,000 rpm at 4 ºC for 30 min, followed by washing with deionized water at least three times and then freeze-dried until an equal dry weight was obtained.

### Catalytic hydrolysis of agar for reducing sugar production

For the production of the reducing sugars from agar, 1% of agar was hydrolyzed with the synthesized Fe_3_O_4_-MPNs in 250 mL of a flask and incubated at 30ºC while shaking at 200 rpm. For enhancing the production of the reducing sugars, different concentrations of Fe_3_O_4_-MPNs (0.1–1%) at various temperatures (30–60 ºC) with different mixture solutions were monitored (dH_2_O, dH_2_O + H_2_O_2_, only H_2_O_2_, 0.1 M and 0.5 M citrate buffer with varying concentrations (100–500 mg/L) of H_2_O_2_). After hydrolysis, the solution was recovered by centrifugation at 13,000 rpm for 30 min at 4 ºC, and the release of the reducing sugar was measured using the 3,5-dinitrosalicylic acid (DNS) (Sigma-Aldrich) method [61].

### Optimization and experimental design

A central composite design (CCD) with independent variables was applied to optimize the significant parameters for the production of bioethanol using the RSM [Design Expert (version 13)]. To determine the individual and interaction effects associated with the process parameters, a CCD factorial design was employed [62, 63]. For error reduction and the uncontrolled factor effects on the response variables, the experimental response was randomized. Following the experimental design, the range and level factors were altered. The response prediction of the correlation and independent variables is illustrated in Eq. 3 as follows:

3 $$Y=f\left(X1, X2, X3\dots \dots \dots \dots .\mathrm{ Xn}\right)+ \varepsilon $$

where *Y* represents the response, *X*_1_ to *X*_*n*_ indicates the independent variables, and ‘ε’ denotes an experimental error. The actual values of the factors and their corresponding coded levels are listed in Table 6. Likewise, − *α*, − 1, 0, + 1, and + *α* indicate five coded levels and their respective values. Using CCD, three different variables (*X*_*1*_: hydrolysate, *X*_*2*_: temperature, and *X*_*3*_: time) were created to develop the experimental runs. To determine the tentative bioethanol concentration, separate experiments were conducted depending on the response variables (Table 1). Based on Table 6, the effects of the different hydrolysate concentrations (1.48%, 10%, 22.5%, 35%, and 43.52%) on the production of bioethanol were estimated. In addition, various temperatures [27.61, 30, 35.5, 37, and 39.39 ºC for *Escherichia coli* K12 and 26.59, 30, 35, 40, and 43.41 ºC for *Saccharomyces cerevisiae* S288c (ATCC 7754)] and times (1.3, 3, 5.5, 8, and 9.07 h) were evaluated to determine the optimum bioethanol production. The analysis of variance (ANOVA) was used to estimate the significance of each factor in the model. Linear, quadratic, and interaction coefficient analyses in terms of the *F* and *p* values are shown in Table 2, demonstrating the significance of the individual and correlated process parameter effects through the response variables. The interaction effect of the two independent variables on the production of bioethanol was visualized using a 3D surface plot.

**Table 6:** Experimental range and levels used in central composite design matrix for ethanol production

Independent variables	Range and level					
	Symbol	− *α*	− 1	0	1	+ *α*
(a) *Escherichia coli* K12						
Hydrolysate concentration (%)	*X*_1_	1.48	10.00	22.5	35.00	43.52
Temperature (^o^C)	*X*_2_	27.61	30.00	33.50	37.00	39.39
Time (H)	*X*_3_	1.30	3.00	5.50	8.00	9.07
(b) *Saccharomyces cerevisiae* S288c (ATCC 7754)						
Hydrolysate concentration (%)	*X*_1_	1.48	10.00	22.5	35.00	43.52
Temperature (^o^C)	*X*_2_	26.59	30.00	35.0	40.00	43.41
Time (H)	*X*_3_	1.30	3.00	5.50	8.00	9.07

### Statistical analysis and model fitting

RSM optimization can be categorized into three major phases: statistical design of experiments, evaluation of coefficients in mathematical models, and validation of the model design [62, 63]. The following equation indicates the calculation of the coded values of the variables from the actual values of the variables:

4 $$Xi=\frac{Xi-Xo}{\Delta {\text{Xi}}}$$

where *i* = 1,2,3……., and *Xi* denotes the variable-coded values *X*_i_; *X*_i_ and *X*_*0*_ represent the actual independent variable values at the center point, and $\Delta Xi$ represents the increment [64].

Individual experiments were conducted for each experimental run to obtain the potential of the bioethanol production, as illustrated in Table 1a, b. To calculate the coefficients for both the individual and combined impacts of the factors, the experimental data were fitted to the following quadratic model:

5 $$Y={\beta }_{0}+{\sum }_{i=1}^{n}\beta iXi+ {\sum }_{i=1}^{n}\beta ii{xi}^{2}+ \sum_{i<j}^{n}{\sum }_{j}^{n}\beta ijXiXj+ \varepsilon $$

Here, *Y* denotes the measured response variable (percentage of bioethanol production), *n* is the number of independent variables, *X*_*i*_ and *X*_*j*_ represent the coded independent variables, and *β*_*0*_, *β*_*i*_, *β*_*ii*_, *β*_*ij*_ are the regression constant, linear coefficient, quadratic coefficient, and interaction coefficient, respectively. *ε* represents the random error estimated from the difference in the observed and experimental values [65].

### Saccharification and fermentation of pretreatment agar

The saccharification of agar hydrolysis was micro-aerobically conducted using *Saccharomyces cerevisiae* S288c (ATCC 7754) and *Escherichia coli* K12 in 100 mL of YM and LB broths containing different concentrations of pretreatment agar solution, respectively. After an equal interval of time, a 1 mL reaction sample was aliquoted to estimate the concentration of bioethanol.

### Analytical methods

**Fig. 12:**
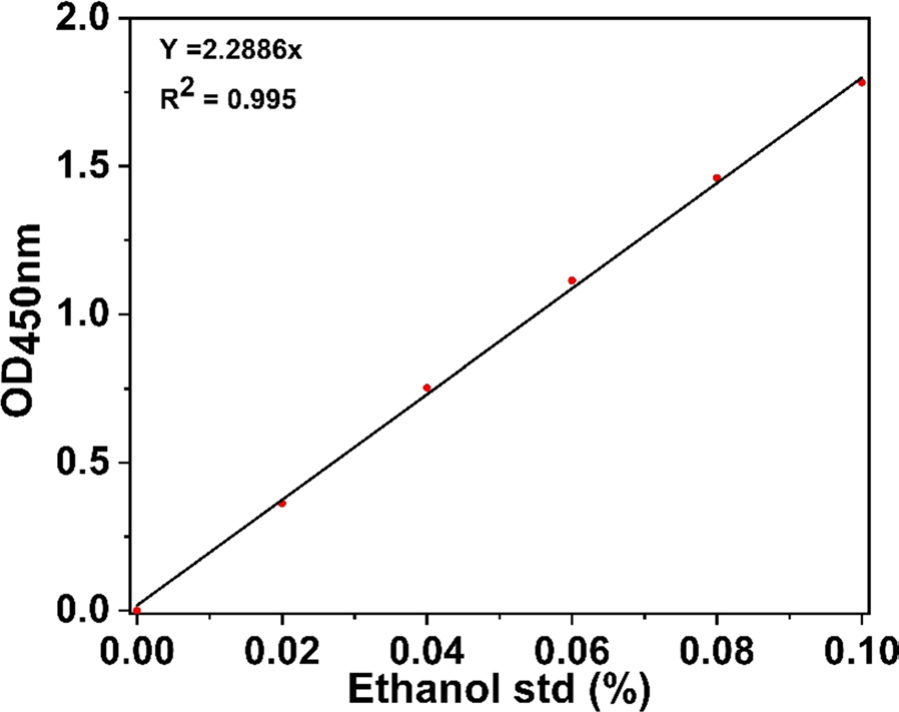
Ethanol standard curve

The bioethanol concentration was measured using an ethanol assay kit (PicoSens™ Ethanol Assay Kit (Colorimetric), Biomax) following the manufacturing company. After centrifugation, the reaction solution was diluted 1:1 with 1X PBS buffer (NaCl, KCl, Na_2_HPO_4_, and KH_2_PO_4,_ pH 7.4). An equal volume of ethanol assay mixture (a mixture of NAD, ethanol probe, enzyme mix, and assay buffer) was added to each well and incubated for 30 min at room temperature. Stop solution was then added to each well, and the absorbance was measured at a wavelength of 450 nm. The bioethanol concentration was calculated by correlating it with the ethanol standard curve (Fig. 12). All experiments were performed in triplicate.

### Model validation

For model validation, the predicted optimum values of the different parameters from the CCD were employed in independent experiments. The experiments were performed at least three times to ensure the reproducibility of the results.

### Characterization of nanoparticles and agar

Fe_3_O_4_-MNPs and agar were characterized using different techniques, such as FTIR spectroscopy (Agilent Technologies (Cary 610/660), XRD [Bruker (D2 Phaser)], SEM (HITACHI (SU3800), TEM (JEM-2100 F HR, Jeol LTD), EDS [JEOL (JSM-6700F/JEOL)], and TGA [TA (SDT 650)].

FTIR was used to determine the major functional groups [66]. The sample was finely ground by mixing it with KBr at a ratio of 1:100 and subsequently pressed to form a thin layer of transparent pellets. Observations were recorded in the range of 4000–5000 cm^2^. Similarly, XRD was conducted to characterize the crystallinity of the synthesized Fe_3_O_4_-MNPs and agar applying the following conditions: cu-Kα radiation (λ = 1.540593 A°) in the 2*θ* range of 5–70° with a stepwise change of 0.0200°. A specific voltage of 45 kV and current of 200 mA were fixed during the analysis. SEM and EDS were used to characterize the surface morphology of the synthesized Fe_3_O_4_-MNPs and agar [67]. Thermogravimetric profiles of the synthesized Fe_3_O_4_-MNPs and agar before and after treatment were determined using TGA. Nearly 10 mg of sample was placed inside the TGA instrument, and the weight loss was constantly measured as the temperature increased from 30 to 900 °C at a controlled heating rate of 10 °C/min under an inert environment (nitrogen gas) without oxygen [57].

## Conclusion

This study emphasizes the future prospects of utilizing abundantly available renewable algal biomass for bioethanol production. The use of the synthesized Fe_3_O_4_-MNPs for agar hydrolysis is faster, simpler, and more economical considering NP application. Furthermore, process optimization using RSM was conducted for comparative bioethanol production, where the agar hydrolysate formed after NP-treated agar demonstrated a significant efficiency in agar degradation. The studies reported thus far have employed simultaneous saccharification using enzymatic hydrolysis and demonstrated a relatively higher ethanol production; however, certain unsolved aspects remain to be addressed. In contrast, the ethanol yield achieved in this study was comparatively lower than those reported, but can overcome the drawbacks of the several other pretreatment methods. In summary, NP-treated agar hydrolysis can be commercialized as a novel method for ethanol production, for which further strategies can be applied to enhance the production rate.

## Data Availability

Available on request.
